# Impact of Abiotic and Biotic Environmental Conditions on the Development and Infectivity of Entomopathogenic Nematodes in Agricultural Soils

**DOI:** 10.3390/insects15060421

**Published:** 2024-06-05

**Authors:** Joanna Matuska-Łyżwa, Sandra Duda, Dominika Nowak, Wiesław Kaca

**Affiliations:** Department of Microbiology, Institute of Biology, Faculty of Natural Sciences, Jan Kochanowski University in Kielce, 7 Uniwersytecka St., 25-406 Kielce, Poland; sandraduda24@onet.pl (S.D.); domi334@o2.pl (D.N.); wieslaw.kaca@ujk.edu.pl (W.K.)

**Keywords:** parasites of insects, soil environment, biopesticides

## Abstract

**Simple Summary:**

Entomopathogenic nematodes (EPNs) are beneficial soil fauna that participate in the food chain. Natural populations of these animals keep insect numbers at a certain level and provide food for other groups of invertebrates. EPNs are also used as biological pest control agents. The life and effectiveness of EPNs are affected by many biotic and abiotic factors of the soil environment. Our review describes characteristics and directions of the effects of various environmental factors on the biology of EPNs.

**Abstract:**

Many organisms, including beneficial entomopathogenic nematodes (EPNs), are commonly found in the soil environment. EPNs are used as biopesticides for pest control. They have many positive characteristics and are able to survive at sites of application for a long time, producing new generations of individuals. The occurrence of populations depends on many environmental parameters, such as temperature, moisture, soil texture, and pH. Extreme temperatures result in a decrease in the survival rate and infectivity of EPNs. Both high humidity and acidic soil pH reduce populations and disrupt the biological activity of EPNs. Nematodes are also exposed to anthropogenic agents, such as heavy metals, oil, gasoline, and even essential oils. These limit their ability to move in the soil, thereby reducing their chances of successfully finding a host. Commonly used fertilizers and chemical pesticides are also a challenge. They reduce the pathogenicity of EPNs and negatively affect their reproduction, which reduces the population size. Biotic factors also influence nematode biology. Fungi and competition limit the reproduction and survival of EPNs in the soil. Host availability enables survival and affects infectivity. Knowledge of the influence of environmental factors on the biology of EPNs will allow more effective use of the insecticidal capacity of these organisms.

## 1. Introduction

The continuing growth of industry worldwide has resulted in an increase in environmental pollution. These developments have had a highly negative effect on beneficial soil organisms. Research carried out so far shows that it is mainly heavy metals, petroleum derivatives, and insecticides that affect these organisms. At high concentrations and after a long exposure time, they can adversely affect beneficial soil organisms.

Within the soil environment, various forms of co-existence occur among different organisms. One of them is parasitism, which is an antagonistic form of dependence. This means that one of the species that participates in the interaction loses while the other gains. We recognize as parasites species that have an intimate long-lived relationship with their host and which benefit from food absorbed from their host, which provides the place of existence for the parasite and its development, which often adversely affects the condition (including the reproductive potential) of the host. Parasitism is common in nature, and parasites include those species that are considered pathogens to plants and animals and to humans [1,2].

Entomopathogenic nematodes (EPNs) have been shown to be highly effective biological control agents with unique advantages over chemical and other biological pesticides. EPNs can kill insect pests within several days of infestation. Tens to hundreds of thousands of new EPNs can be produced from a single infested insect host and then travel to infest new hosts, thus theoretically allowing EPNs to persist in the field throughout the growing season [3,4]. EPNs are compatible with most other agrochemicals, with which they can be co-applied [5,6,7,8,9,10,11], and have minimal undesirable side effects on nontarget organisms. This is the case with chemical pesticides, which lead to soil contamination and bioaccumulation [4,12]. Despite these advantages, EPNs are still rarely used in agriculture due to costs and the sensitivity of EPNs to environmental factors [13].

The article aims to characterize the use of entomopathogenic nematodes (EPNs) for biological pest control, present the development and parasitism strategies of EPNs, describe the optimal environmental conditions for EPNs and their interspecies relationships, and present the effects of soil contaminants on EPNs (heavy metals, petroleum substances, pesticides, insecticides, and anthropogenic activities).

## 2. Entomopathogenic Nematodes (EPNs) and Biological Pest Control

Entomopathogenic nematodes are a group of parasitic organisms that are part of the natural soil mesofauna and are a factor that naturally controls the density of insect populations. EPNs are also an example of another interesting relationship between organisms—mutualism—that is beneficial to both coexisting species, which are dependent on each other to such an extent that, under natural conditions, they could not exist separately. EPNs exist in this particular kind of symbiosis with the bacteria to which they owe their ability to quickly kill an infested insect [14]. Using the right species of nematodes, pests of various crops can be controlled. This method is an alternative to the use of chemical plant protection products, enabling a reduction in the use of hazardous substances in agriculture or horticulture. Moreover, the isolation of highly virulent EPN strains from local soils removes the need for the introduction of foreign organisms, thus avoiding potential regulatory constraints [15,16]. In addition, EPNs pose no threat to warm-blooded organisms and nontarget organisms; they only attack target organisms [17,18].

Currently, preparations based on insecticidal nematodes are directed against thrips (Thysanoptera), diptera (Diptera), beetles (Coleoptera), and butterflies (Lepidoptera). Research on the effectiveness of EPNs against various pest species has been conducted under field and laboratory conditions [19,20,21,22,23,24,25,26,27]. One of the advantages of using preparations containing these living organisms is that, following a single application, EPN populations can survive at the application site for a long time, producing new generations. Sources say that the duration of action of the first generation introduced into the soil is 6 to as long as 16 weeks. Due to the short development cycle, this is enough time to produce a significant number of offspring. Also important is the fact that, during the process of preparing a biopreparation for spraying, nematode mortality is negligible. As a result, the product does not lose quality, which increases the chances of effective control of crop pests [28,29,30]. Unfortunately, EPN preparations have shortcomings in terms of biological pest control. The main ones are, primarily, the high level of host selectivity exhibited by EPNs, as well as the possibility of their encountering natural enemies in the soil, such as other nematodes or fungi [31]. Commercially available EPN preparations containing infective juveniles (modified third-stage free-living juveniles) are produced in bioreactors, and biopreparations are placed in the soil using tractor or knapsack sprayers, although nematodes can also be introduced into the soil by manual watering for home garden use. Infective juveniles can be passed through spray tubes with a diameter of at least 500 μm and are capable of withstanding pressure levels of up to 2000 kPa [14]. Commercial preparations of nematode juveniles also contain components that improve the persistence of these organisms. An example is the addition of gelling substances that protect nematodes from drying out. Due to these additives, EPNs can perform a protective function for plants for much longer. Gel formulations allow the application of doses of EPNs lower than those commonly used to control underground pests. EPN–gel applications increase crop yields [5,32,33,34,35,36].

## 3. Development and Strategies of Entomopathogenic Nematode Parasitism

Entomopathogenic nematodes probably evolved from free-living nematodes which fed on bacteria [37]. By 2020, more than 100 species of nematodes had been assigned to the *Steinernema* genus (Rhabditida: Steinermatidae), while 21 species had been assigned to the *Heterorhabditis* genus (Rhabditida: Heterorhabditidae) [38]. Each species of nematode coexists with only one species of bacteria, but the same bacterial species can live in symbiosis with different species of EPNs. Each *Steinernema* species is mutualistic with only one *Xenorhabdus* species, but some *Xenorhabdus* species are symbionts of multiple *Steinernema* species. *Steinernema feltiae* Filipjev (Steinernema: Steinermatidae) is related to *Xenorhabdus bovienii* Akhurst and Boemare (Enterobacterales: Morganellaceae), while *X. bovienii* lives in symbiosis with *Steinernema affine* Bovien (Steinernema: Steinermatidae), *Steinernema kraussei* Steiner (Steinernema: Steinermatidae), and *Steinernema intermedium* Poinar (Steinernema: Steinermatidae) [39,40].

Of the different stages of EPN development, only the third-stage infective juvenile (IJ) can live in the external environment, leaving the body of the infested insect host. Therefore, juveniles at this stage are adapted to the conditions they encounter in the soil. These juveniles have a thick and resistant cuticle, and the mouth and intestines are closed [41]. Inside their body, there are fat granules, which act as food reserves, because the IJs do not absorb nutrients from the external environment [28]. Depending on the amount of accumulated fat reserves and the general condition of the juveniles, the search for a host can last for several days. The hosts of EPNs are usually insect larvae, much less often adults, in which the development of nematodes is slower [17,42,43]. The susceptibility of *Tuta absoluta* Meyrick (Lepidoptera: Gelechiidae) adults has been shown by Batalla-Carrera et al. (2010) in preliminary experiments with continuous exposure to EPNs in laboratory conditions [44]. The juveniles at this stage are called infective because they actively search for a host, and, when they encounter a suitable insect host, they penetrate inside it via either the cuticle or natural orifices (e.g., the mouth, anus, or spiracles) of the body [45].

The finding and infestation of an insect occurs thanks to a specialized system of chemoreceptors (amphids) developed in the form of lateral organs in the area of the lips. Infective juveniles react to the presence of a host by moving towards an increasing concentration of chemical signals (chemotaxis) emitted by it, such as CO_2_, components of insect feces (e.g., ammonia, uric acid, and arginine), and other specific compounds [46,47,48]. In addition to chemical signals, IJs also respond to insect movements in the soil (thigmotaxis). Studies have shown that IJs of the genus *Heterorhabditis* show a greater chemotactic capacity than IJs belonging to the genus *Steinernema*, which show a greater thigmotactic capacity [49].

Some EPN species are not very active, staying close to the soil surface and using an “ambush” strategy in which they stand on their tails and wait for passing insects. This is the strategy used by *Steinernema carpocapsae* Weiser (Steinernema: Steinermatidae). It is one of many tactics used by insect nematodes. Other species penetrate more deeply into the soil matrix and use an “active hiking” strategy to locate and infest sedentary insects. “Cruiser” behavior is used by *Steinernema glaseri* Steiner (Steinernema: Steinermatidae), *Steinernema arenarium* Artyukhovsky (Steinernema: Steinermatidae), *Heterorhabditis bacteriophora* Poinar (Rhabditida: Heterorhabditidae), and *Heterorhabditis megidis* Poinar, Jackson and Klein (Rhabditida: Heterorhabditidae), using the “trap” method. EPN species which use both methods of host searching include *S. feltiae* and *Steinernema riobrave* Cabanillas, Poinar and Raulston (Steinernema: Steinermatidae) [50].

The mutualistic relationship between bacteria and EPNs is based on several interactions. Nematodes carry symbiotic bacteria in the intestines of their bodies, allowing them to spread and infest new insects. In turn, bacteria kill the insect quickly, thanks to the production of enzymes and toxins. Bacteria producing these compounds also deter saprophytes from colonizing an infested insect cadaver, making it an unattractive food source for other organisms, as well as providing the nematodes with nutrients [51,52].

In the intestines of infective juveniles there are cells of symbiotic bacteria [53,54]. Bacteria of the genus *Xenorhabdus* are mutualistically related to the nematode genus *Steinernema* and are found in specialized intestinal alveoli [55,56]. *Heterorhabditis* nematodes form a symbiotic system with *Photorhabdus* bacteria, which are stored throughout the intestines of the nematodes [57,58,59]. Before the release of the bacteria, *Steinernema* spp. produce toxins (e.g., *Xpts*, *Txp40*, and *Xax*) that block the immune response of the insect, creating better conditions for the bacteria which will be released later. These toxins also enhance the pathogenic effects of the symbiotic bacteria [45,60,61,62,63]. In the nematode family Heterorhabditidae, no proteins with similar effects have been reported [56].

After penetration by an infective juvenile into the body of an insect host, bacteria are released from the intestines of nematodes into the hemocoel of the insect. Depending on the nematode family concerned, the bacteria are released in one of two ways: members of the Steinernematidae release their bacteria by defecation [64], whereas members of the Heterorhabditidae release bacteria through their mouths [57].

In the hemolymph of a host insect and its internal organs, the bacteria destroy the hemocytes (causing septicemia), multiply, and begin to produce exoenzymes as well as the toxins that destroy the immune system of the insect. These bacterial products cause biochemical changes in the body of the insect host, which leads to its death within 24–48 h [47,56,60,65,66,67,68]. In addition, they produce antimicrobial bacteriocins [69] and antibiotics that inhibit the growth of fungi, yeasts, putrefactive bacteria, and other pathogens (e.g., *Shigella sonnei* Levine (Enterobacteriales: Enterobacteriaceae) and *Flavobacterium* sp. (Flavobacteriales: Flavobacteriaceae) [70,71,72].

As a result of the action of antimicrobial substances produced by the symbiotic bacteria, the body of an infested insect does not decompose. In the initial period of infestation, the body of the host becomes rubbery, darkens over time, becomes stickier, and, finally, disintegrates. Depending on the species of nematode and the bacteria associated with it, the body of an insect after infestation can take on a different color [58,73,74,75,76].

Research on the nematode–bacterium system indicates that, in addition to the main EPN bacterial endosymbionts (*Xenorhabdus* and *Photorhabdus*), there is a frequently associated microbiota (FAM) consisting of several members of the Proteobacteria phylum. The endosymbionts and the accompanying microbiota determine the pathobiome of EPNs [77,78,79].

**Figure 1:**
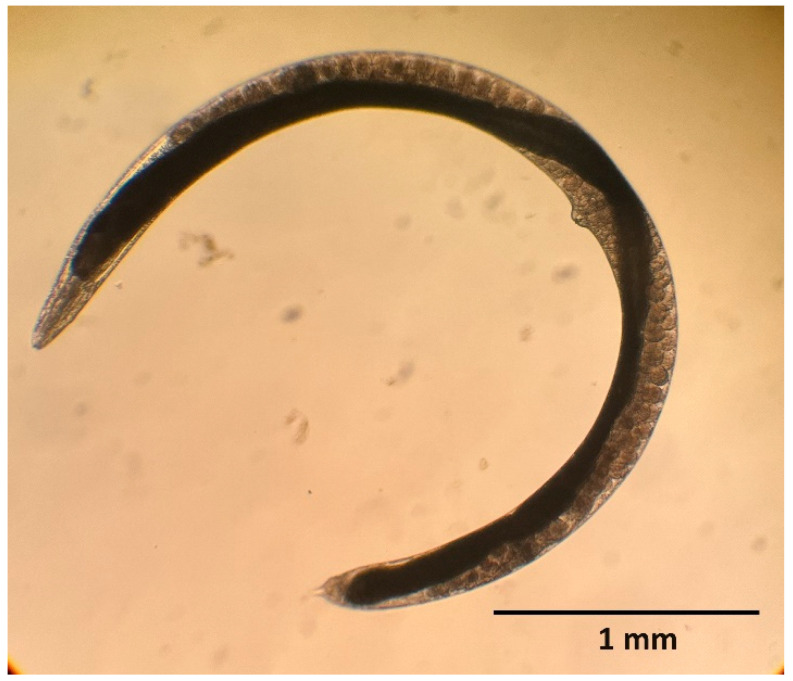
First-generation female of *Steinernema feltiae*.

Infective juveniles (J_3_) that penetrate into the host insect molt to the fourth juvenile stage (J_4_), which is capable of taking in nutrition from the host [17,80]. The developing nematodes feed on dead bacteria, substances produced by the bacteria, as well as insect tissues, altered by the influence of bacteria [74,81]. J_4_-stage nematodes reach adulthood within 2 or 3 days when cultured in *Galleria mellonella* Linnaeus (Lepidoptera: Pyralidae) at 23 °C [82]. In the Steinernematidae, at the J_4_ stage, male and female adults develop, whereas in the Heterorhabditidae, hermaphroditic individuals are formed (in subsequent generations, there are individuals of both sexes). Individuals in the first generation of adults are called giants, due to their larger body size compared to the individuals of generations which arise later. Giant females (Figure 1) reach over 6 mm in length, while males may slightly exceed 3 mm in length [28,81,83]. The number of generations that develop in the body of an insect depends mainly on the amount of food available. Different ways of development can occur depending on the density of nematodes inside the body of the host insect. If the density is low, the females reach larger individual body sizes and lay a large number of eggs, with a proportion of the eggs developing in the body of the female. First-stage juveniles (J_1_), emerging from the eggshells, initially feed on the reproductive system of the female and, after its rupture, on the pseudocoel, leading to her death. This phenomenon of intrauterine birth is called endotokia matricida or facultative vivipary. During this time, the juveniles develop rapidly from J_1_ to J_4_ and from J_4_ to adulthood. When the density of nematodes inside the body of the host insect is high, however, individual females are smaller, and most eggs develop within their bodies [84].

In *H. bacteriophora*, an individual in the hermaphrodite generation lays about 1000 eggs; in the case of *S. carpocapsae*, however, a first-generation female lays about 3000–4000 eggs. Individuals of the next generation of *H. bacteriophora* each lay 3000–5000 eggs, whereas those of *S. carpocapsae* lay 12,000–14,000 eggs [82]. The final number of infective juveniles is similar in both species; in *H. bacteriophora*, −1% of the eggs in hermaphroditic individuals develop only to the J_3_ stage, which then leave the body of the insect host. From the second generation, in which there are females and males, 5–10% of the eggs develop to the J_3_ stage, which also leave the body of the insect host. From the next generation, all eggs develop into J_3_s, which leave the host and enter the external environment. In *S. carpocapsae*, the J_3_ juveniles that leave the body of the insect come from the second and third generations of adults [81]. A multistage population transforms into a monostadial population (infective juveniles) when food begins to run out. At this time, nematodes end their development as a generation of infective juveniles which take into their digestive tract cells of the symbiotic bacteria which multiplied in the body of the dead insect host [58,85]. In these juveniles, the opening anal orifice closes, the mouth regresses, and bacteria are stored in the intestine. Juveniles at this stage are capable of migrating to the external environment and infesting another insect [86]. The length of the development cycle, from the penetration of juveniles into the body of the insect to the exit of the next generation, in the Heterorhabditidae is 10 to 16 days, and in the Steinernematidae it is 10 to 14 days [82].

If suitable conditions prevail outside the body of the host, the juveniles leave the body. However, if the humidity or temperature is too low, the juveniles can stop migrating to the external environment for up to 50 days. The juveniles also have the ability to enter diapause, in which they can remain for up to 3 years [87,88]. During the free-living phase of the nematodes, their symbiotic bacteria stay active at all times. The greater the metabolic activity of the bacteria, the shorter the period for which the infective juveniles of the nematode can live in the external environment. This is due to the limited amount of nutrients accumulated in the body of the nematode. However, when a host is infested by the nematode, the high activity of bacteria has positive effects because they lead to faster insect death. Because of the possibility of the premature death of the nematode in the absence of a host, it is beneficial to maintain metabolic activity at an optimal, moderate level [89,90,91].

Insects have all kinds of defense mechanisms. The first of these is behavioral protection, e.g., intensive defecation in beetle larvae significantly hinders the penetration of nematode juveniles through the opening in the beetle larvae [92]. Another strategy is mechanical defense in the form of a protective cuticle on the surface of the body. However, if a nematode juvenile gets inside a host larva, the mechanisms of the host’s immune response are activated. The cellular response involves hemocytes, immune cells that are responsible for phagocytosis but which can also perform other complex functions, such as surrounding and encapsulating parasites like the nematode. The humoral response includes the production of antibacterial peptides. One of the functions of the peptides is to interact with hemocytes in the activation of phenoloxidase, an enzyme involved in the activation of metabolic pathways that lead to the neutralization of pathogens. This enzyme induces the production of toxins and products involved in encapsulation. Other defense strategies include the production of reactive oxygen and nitrogen species [51,93,94,95,96].

EPNs have adapted in response to the harsh conditions they encounter in the bodies of attacked host insects. They have developed mechanisms that make it possible to bypass or even neutralize the host’s defense factors. It is possible, in principle, to achieve the biochemical masking of the nematode from the insect’s immune system. In turn, on the surface of the cuticles of nematodes, there are molecules which block the secretion of antibacterial peptides from the host. These nematodes also secrete substances that kill hemocytes. An example is *S. feltiae*, which has lipopolysaccharides (LPSs) in its cuticle that inhibit the production of the phenoloxidase enzyme [94,97].

Entomopathogenic nematodes exhibit intraspecies variability, which is related to their location and adaptation to a particular habitat. This diversity can be manifested in differences in developmental cycles, reproductive strategies, and virulence toward host insects. Examples of intraspecific diversity could be seen in *S. feltiae* isolates collected from different habitats in northern Spain. Not only did the nematodes themselves show differences, but the associated bacteria did also. Their functions differed between populations of different origins [98,99,100].

Another example of intraspecies variability is the differential tolerance to high temperature and low humidity in representatives of the genus *Heterorhabditis*. During the study, it was observed that, within the same species, there are individuals that are more or less tolerant to stress conditions. Genetic mechanisms are responsible for this diversity, which allows populations inhabiting hot and dry areas to develop adaptations necessary for living in such environments [101].

## 4. Composition of Soil and Its Effect on Nematode Populations

Entomopathogenic nematodes occur in both natural and anthropogenic ecosystems, where they shape biological diversity. The occurrence of certain nematode species depends on their nutritional preferences and, consequently, on the presence of specific host insect species in these locations [102].

Research on the distribution of EPNs showed that the presence of nematodes of the Steinernematidae family is commonly reported in forested areas [102,103,104,105,106]. *S. kraussei* and *S. intermedium* are most commonly isolated from forest areas, with the former begin found mainly in coniferous forest soils of Europe and North America [105,106]. Areas transformed by humans also create suitable living conditions for these organisms. *S. feltiae* has been shown to be a recorded species in meadows, forests, and agricultural fields [105,107,108], while *S. affine* and *S. carpocapsae* have also been found in cultivated soils [105,107].

Nematodes of the family Heterorhabditidae are often associated with sandy coastal soils and grasslands [104,107,109,110], the most commonly isolated species being *H. megidis* [107,111,112,113].

**Figure 2:**
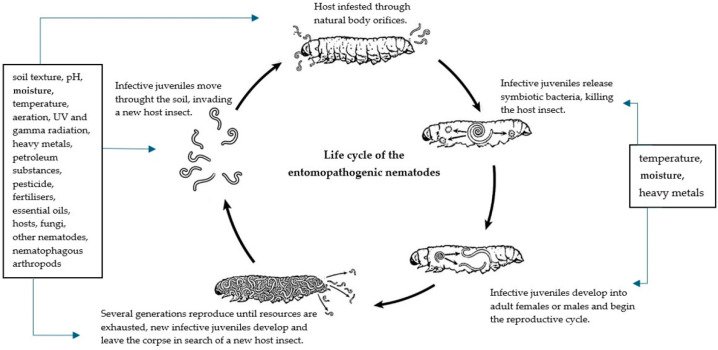
Factors influencing the development of entomopathogenic nematodes.

The distribution of EPN populations in a particular area depends on many environmental factors, such as soil moisture, temperature, structure, and pH [114]; the availability of hosts; and the presence of competitive or predatory organisms [115,116,117] (Figure 2).

### 4.1. Soil Texture

The mechanical composition of soil, e.g., grain size, affects soil organisms and other conditions of the soil environment, such as the degree of soil moisture and aeration. Lighter soils are better aerated and are more easily permeable to lower layers. Heavier soils have a greater water-carrying capacity, but air circulation is poorer. Individual species of EPNs differ in their requirements in terms of air–water relations in the soil environment. Soil structure influences, among other things, the movement, infectivity, and survival of nematodes [118,119,120,121,122]. Depending on the size of the soil fraction, the rate of movement and the frequency of undulating movements changes. In heavier soils, infectivity and movement are hindered, whereas in light loam soils with similar percentages of silt and clay, they are facilitated [48,99,121,123,124]. Other research has shown that, in soil composed of about 40% sand, 33% silt, and 26% clay, nematodes moved at a rate of 4.35 cm/day and covered a distance of 46 cm in 14 days. In sandy soil with a mean grain diameter of 0.2–0.5 μm, infective juveniles covered a distance of 2 cm (87%) within 48 h; 0.5% of the infective juveniles covered a distance of 12–14 cm from the site of release [125].

Research into vertical migration showed that it decreases in response to the increase in clay and silt content, with sandy soil making it easier for nematodes to move in this direction [126]. Most nematode species prefer to migrate upward rather than downward. One example is the species *H. bacteriophora*, which infects insects above the EPN application site [127]. Other species, such as *S. carpocapsae*, prefer to migrate downward; from the site of application of the infective juveniles, they move deep into the soil, where they effectively infest host insects [128]. The reason for such different directions of movement is the difference in the rate of penetration of signal chemicals secreted by hosts that attract the nematodes [129].

The structure of the soil also affects the infectivity of EPNs [130]. Juveniles have been shown to penetrate hosts more quickly and willingly in sandy and sandy loam soils [48]. The highest infestation levels were obtained in clay–sandy soils with low moisture and in sand with high relative moisture, close to saturation. It was shown that, as the clay content of the soil increased, the infectivity of *S. glaseri* decreased [131].

Soil texture is also one of the most important factors influencing nematode survival [123,132]. In research on *S. carpocapsae* and *S. glaseri*, infective juveniles of these species placed on sterile sandy, sandy loam, or loam soils for 16 weeks showed different degrees of survival. The lowest survival rate was associated with the loam soil because the low porosity and poor aeration of this type of soil limited the survival rate of infective juveniles. Similar conditions prevailed in clay soils, which also reduced the effectiveness of the EPNs. On the other hand, the highest survival rate was recorded in sandy loam and sandy soils. It was shown that different EPN isolates can show different adaptations to the textural classes of soil. Native isolates may show better performance in the environment from which they originate [48,121,133,134].

### 4.2. Moisture

The level of moisture in the environment in which EPNs are located affects their survival rate, with the appropriate moisture level making it easier for them to move around and search for a host. The movement of nematodes within soil pores is enabled by a thin water capsule around the body of the nematode [14,135,136,137].

The type and rate of nematode movement depend on the thickness of the water capsule: the thicker the capsule, the greater the distance the infective form travels [129]. In a dry environment, movement practically stops, and individuals either enter a state of anabiosis or die [124].

High soil moisture also adversely affects the ability of nematodes to move [138]. This is due to the lack of surface tension necessary for movement [139]. Research on infective juveniles has shown that, by making pendulum movements on the soil surface at a moisture content of 25–40%, juveniles have the most favorable conditions for invasion of the host. When the soil moisture content exceeds 50%, the biological activity of infective juveniles is limited [48]. The infectivity of nematodes is also reduced when the soil environment is too dry [140].

Soil moisture also affects nematodes living inside a host insect’s body. In dry soil, a small number of juveniles were observed leaving the host body. At low moisture, *S. glaseri* and *S. carpocapsae* juveniles leave the body of the host insect the earliest, whereas *S. riobrave* juveniles leave the insect body the latest [141]. The reason for this phenomenon may be the inability of the nematodes to physiologically adapt to the low moisture conditions of the soil environment after abruptly leaving the host organism. Under such unfavorable conditions, the juveniles can survive for between a few hours and several days, as they are exposed to rapid drying [142]. On the other hand, when the moisture is low, the juveniles cannot stay in the body of the host for too long, as the cuticle of the insect begins to dry out and harden, making it difficult for the juveniles to leave the body of the insect [141].

### 4.3. Temperature

Another factor influencing the biology of EPNs is temperature. The degree of temperature tolerance varies from species to species and even between strains of the same species [110,143]. A common feature of temperature response is the tendency for nematode survival to decrease with increasing temperature. Nematodes are less sensitive to low temperatures than to high temperatures [143,144,145]. Nematodes that are in anabiosis are more tolerant of extreme temperatures than nematodes that are active under these unfavorable conditions [124]. Nematode strains that are used in the biocontrol of pests of living plants are active at temperatures between 10 °C and 30 °C [146,147,148].

The optimal temperature for infestation by different species of the Steinernematidae is in the range of 13–20 °C [149]. Studies have shown that the optimal temperature for *S. carpocapsae* is in the range of 20–25 °C. These studies also showed that, at 10 °C, only fourth-stage juveniles (J_4_) were present in the dead host (*G. mellonella*, the greater wax moth). The full development of the nematodes occurred at a temperature of 15 °C, but no migration of the juveniles to the external environment was found at that temperature. When the temperature rose above the optimal value (30 °C), it was shown that only first-stage juveniles were present in the insect host [150]. Research on temperature-dependent infectivity showed that *S. glaseri* and *S. feltiae* showed a greater infestation of insects at 25 °C than at 15 °C, although no significant differences was observed for *S. arenarium* [149,151,152]. Similar studies were conducted for *H. megidis*, where the highest infectivity and fastest development were recorded at 25 °C [153], although soil moisture had to be acceptable. The activity of *H. megidis* juveniles decreased when the ambient temperature fell to 15 °C. As the temperature increased, these juveniles regained their ability to attack and kill the insect host [124].

In the case of extreme low temperatures, nematodes have been shown to survive temperatures below 0 °C. In the case of unencapsulated nematodes, juveniles freeze at −6 °C, while *Heterorhabditis zelandica* Poinar (Rhabditida: Heterorhabditidae) is encapsulated and can survive at −32 °C [154]. Other species also exhibit high tolerance to low temperatures. Examples include *S. feltiae*, *S. arenarium*, and *H. bacteriophora*, which can survive at −22 °C, −14 °C, and −19 °C, respectively [155].

High temperatures above 32 °C also adversely affect the infectivity, reproduction, growth, and survival of nematodes [146,156]. It has been shown that storing *S. feltiae* at 35 °C for one hour caused a dramatic decrease in activity, whereas storing them at 37 °C for 16 h led to the death of all infective juveniles [157,158].

Some strains of EPN species from the Heterorhabditidae are also tolerant to high temperatures. This is evidenced by the isolation of these nematodes from hot and dry areas. Such examples have been isolated from Egypt, Sri Lanka, Israel, and Iran [159,160,161,162].

### 4.4. Soil pH

Soil pH is a key element that affects many biological and physicochemical processes in soils. The degree of soil acidification largely determines the mobility and bioavailability of heavy metals, as well as ionic organic pollutants. With an increase in acidification of the soil environment, the bioavailability of heavy metals increases. The optimal pH range of soil for the growth and reproduction of most plant species and soil organisms is considered to be between 5.5 and 7.2 [163].

In the case of EPNs, acidic soil pH levels cause decreases in juvenile infectivity and survival. The survival of *S. carpocapsae* and *S. glaseri* was reported to be similar at pH 4, 6, and 8 for the first 4 weeks. Storage of these species for 16 weeks in an environment with a pH of 10 showed that the survival rate of *S. carpocapsae* was significantly greater than that of *S. glaseri* [164]. *S. feltiae* and *H. bacteriophora* showed greater mobility, activity, and pathogenicity at pH 6.8 and 8 than at pH 5.5 [165].

### 4.5. Radiation

An important factor influencing EPNs is ultraviolet (UV) and gamma radiation. Understanding the effects of UV radiation is important when using nematodes to control pests found on the above-ground parts of plants. It has been shown that 30 min of exposure of nonencapsulated nematodes to sunlight reduced their reproduction and increased their mortality [166]; encapsulated nematodes were not affected as much. The researchers also showed that exposure to long-wavelength (366 nm) UV radiation for up to 8 h did not have a negative effect on infective juveniles, unlike the response to short-wavelength UV (254 nm). Exposure to short-wavelength UV radiation for 1.75 min reduced the pathogenicity of infective juveniles by 19%, and exposure for 7 min reduced pathogenicity by 100% [166]. In viability assessments, *Steinernema* species, especially *S. carpocapsae*, showed greater tolerance to UV radiation than *Heterorhabditis* species, *H. megidis* and *H. bacteriophora* being the most sensitive [167,168].

Research on the effect of gamma radiation on entomopathogenic nematodes showed that infective juveniles of *Steinernema scapterisci* Nguyen and Smart (Steinernema: Steinermatidae) and *H. bacteriophora* irradiated with 2 Gy gamma radiation were more effective in biocontrol of Egyptian cotton leafworm, *Spodoptera littoralis* Boisduval (Lepidoptera: Noctuidae), than were nonirradiated nematodes. Similar results were obtained for *S. carpocapsae* BA2 [169,170].

### 4.6. Soil Aeration

Soil aeration and compaction also have a major impact on the development of EPNs. The oxygen and carbon dioxide concentrations of the water molecules in which the infective juveniles are present depend on aeration. EPNs are aerobic organisms, and a low oxygen content may reduce their survival in the soil environment [171,172]. The air content of the soil is variable and depends on the soil type. Heavy moist soils are conducive to anaerobic conditions, which, in most cases, are harmful to nematodes. Factors that are negatively related to the oxygen content of the soil environment include the contents of clay and organic matter [48]. The percentage composition of gases in dry sandy soil is similar to that in the atmosphere, so such soils create conditions conducive to nematodes [124]. Despite oxygen being present in soil clods, nematodes only use the oxygen dissolved in the water surrounding the clods. The amount of oxygen depends on the spaces between the clods, the type of soil, and the degree of water saturation [124].

Research shows that the respiration rate of *S. carpocapsae* depends on the ambient temperature [173]. It was also shown that low O_2_ concentrations interfered with pathogenicity and led to the death of nematode juveniles. The survival rate of *S. carpocapsae* and *S. glaseri* infective juveniles in sandy loam soils decreased in response to a decrease in oxygen content from 20% to 1% [164,173]. Infective juvenile forms of EPNs use elevated carbon dioxide concentrations as signals with which to locate a potential host [48].

## 5. Soil Contaminants Affecting Entomopathogenic Nematodes

Soil is a natural habitat for the development of many organisms that condition the decomposition and production of biomass. The soil environment filters groundwater and also ensures the circulation of matter and the flow of energy within the ecosystem. Soil also buffers, filters, and accumulates harmful substances. Many factors contribute to increases in soil pollutants, such as industry, transport, and improper sewage and waste management [174].

### 5.1. Metal Ions

Heavy metals are potential contaminants resulting from anthropogenic pollution. They enter the environment from various sources [175,176]. These pollutants are very harmful to the biologically active layer of the soil; because the metals are not susceptible to leaching, they persist in the soil environment for a long time, limiting the suitability of the soils for agriculture. Excessive accumulation of heavy metals in the soil negatively affects the organisms present. In many cases, these compounds accumulate in the organisms, leading to disorders in metabolism and changes in the structure and function of cells and tissues [177,178,179]. The toxicity of heavy metals and their high bioavailability depend on many factors, such as the amount of contamination and the chemical form, as well as on the properties of the soil environment: temperature, pH, presence of other metals, and oxidation–reduction potential [180,181].

In the soil environment, infective EPN juveniles will be directly exposed to any heavy metals found there. These compounds affect the occurrence, survival, infectivity, and reproductive capacity of nematodes. Natural concentrations of heavy metals in the habitats of nematodes are not the cause of their mortality. On the other hand, they are the reason for the reduced infectivity of EPNs. The most adverse effects were observed with nickel (Ni(II)) and lead (Pb(II)) ions [182,183]. Laboratory studies on the effect of heavy metals on EPNs have shown that heavy metal ions can adversely affect the pathogenicity of EPNs [107,180,182,183,184,185,186,187,188,189,190].

In the first days of contact with infective *S. feltiae* juveniles, low concentrations of Pb(II) and cadmium Cd(II) ions in the sand cause an increase in the intensity of invasion of the host. This is caused by a stress response that stimulates the nematodes to quickly invade the body of the host. In soils with a higher concentration of heavy metal ions, negative parameters, such as lower infectivity, increased mortality of nematodes inside the body of the insect host, and increased mortality of individuals migrating in the external environment, have already been shown to occur in a metal ion-concentration-dependent manner [187,191].

Heavy metals also hinder the movement of nematodes. Studies on the effects of metal salts showed that the presence of these compounds in soil prevented nematodes from moving, finding, and infesting hosts. Chloride salts have been found to be particularly toxic [192]. In subsequent analyses, it was determined that the addition of magnesium salts to solutions in which nematodes were stored had a protective effect against heavy metal ions [191,193,194].

Research into the effects of combinations of several metals on *S. carpocapsae* showed that these combinations reduced the pathogenicity of EPNs. The presence of lead, cadmium, and copper in the soil resulted in the nematodes killing a smaller number of host insects compared to the control samples (in natural, unadulterated soil). The addition of magnesium to soil contaminated with heavy metal ions significantly increased the number of nematodes entering hosts [190]. Different combinations of metal ion triads were also created in the study, based on Cd(II), cobalt (Co(II)), copper (Cu(II)), magnesium (Mg(II)), manganese (Mn(II)), Pb(II), and zinc (Zn(II)). In most cases, the nematodes tolerated the toxicity of heavy metals. On the other hand, Cd(II) and Pb(II) ions always killed the nematodes. None of the added metal ions reduced the toxicity of Pb(II), while the toxicity of Cd(II), together with Cu(II), Zn(II), and Co(II), was reduced by the addition of Mg(II) but not Mn(II). The toxicity toward *S. carpocapsae* of aqueous solutions of selected triads of metal ions depended on the method of introduction of these ions into the nematode environment. Mg(II) ions showed a protective effect over a long period of time or induced rapid death of nematodes. Mn(II) ions, on the other hand, provided short-term protection, with generally low toxicity [180].

Insect nutrients contaminated with Pb, Cd, or Cu ions also had an adverse effect on infectivity, nematode development in the host, and the viability of juveniles migrating from the host into the environment. These ions accumulate in the tissues of the insect host, which indirectly affects the nematodes [195,196,197].

Heavy metals affect not only EPNs, but also the associated bacteria present in their digestive tracts. It was shown that copper, manganese, and nickel ions were most harmful to these microorganisms. In contrast, lead was not toxic to nematode-associated bacteria [198].

The characteristics of the soil environment in which EPNs occur are also influenced by human activities. These include chemical or physical changes to the soil caused by harrowing or application of fertilizers, pesticides, insecticides, or fungicides [199].

### 5.2. Petroleum

Petroleum derivatives found in agricultural soil destabilize the biological properties of soils, as well as the biotic community, and finally affect the quality and fertility of the soil. Petroleum substances that may adversely affect the development and function of EPNs are mainly crude oil, diesel oil, engine oil, and gasoline (“petrol”). Of the above-mentioned compounds, gasoline is the least harmful, and engine and diesel oils are the most harmful [200]. All these substances reduce the pathogenicity and reproductive capacity of EPNs. The longer the contaminant is active, the higher the mortality of the nematodes. Mortality is also related to the occurrence of EPNs in land contaminated with petroleum substances. EPNs could not be isolated from soil contaminated with petroleum derivatives [201]. In addition, these compounds inhibit the ability of EPNs to find a host [202] and reduce their infectivity and reproduction [203,204].

### 5.3. Pesticides

Among the pesticides used in plant protection, products containing oxamyl, sulfur, mancozeb, and fenitrothion turn out to be highly toxic to nematodes. Trifluralin causes a high mortality rate in EPNs. Pesticide concentration has also been shown to have a major impact on the infectivity of EPNs [205].

In research into the effects of various pesticides on EPNs, it was shown that most fungicides, pyrethroid insecticides, and chlorinated hydrocarbons were nontoxic or showed low toxicity toward *S. feltiae* larvae. These compounds also did not reduce the infectivity of the EPNs. In the case of urea and carbamine herbicides, the viability of infective juveniles decreased significantly, and those that survived contact with herbicides were characterized by low infectivity. The ability to find and infect a host was also impaired. In an environment in which herbicides were present, female EPN reproduction was limited. Within 5 months of the application of triazine, diazine, and uracil herbicides, there were no differences in the numbers of nematode juveniles harvested from the soil and capable of infesting insects [206].

Similar effects were observed for organophosphate compounds, chlorinated hydrocarbons, diflubenzuron (an insecticide from the group of acylurea compounds), and synthetic pyrethroids. These agents adversely affected nematodes in in vitro culture, while methomyl reduced the reproduction of *S. carpocapsae* [207,208].

Insecticides are also used as protection products against pests in animals. Such an agent is fipronil, which controls fleas and ticks in dogs, poultry red mites, and ants, cockroaches, flies, and mosquitoes. Another example is malathion, an agent effective in the treatment of head lice, scabies, and poultry lice. These insecticides were found to have no adverse effects on the biology of *S. carpocapsae* [209].

Insecticides can also be isolated from plants. An example is azadirachtin, extracted from *Azadirachta indica* A. Juss. (Sapindales: Meliaceae) (the neem tree). It is effective against mealybugs (Hemiptera: Pseudococcidae), aphids (Hemiptera: Aphidomorpha), and *L. decemlineata* and is safe for birds and beneficial insects. It was shown that this agent does not adversely affect EPNs, is toxic only at doses higher than recommended, and can be used in combination with EPN-based insecticides [210,211].

Insecticides are chemical agents directed against plant insect pests. They are used as stand-alone preparations or in combination with biological methods in Integrated Pest Management (IPM). However, they can affect the biology of EPNs. One group of insecticides which can be used together with EPNs are the neonicotinoids [212]. Imidacloprid was shown to have no adverse effect on EPN pathogenicity or the infectivity of juveniles and can be used in combination with EPN-based preparations [209,212,213,214]. However, at high doses, similarly to acetamiprid, it causes a decrease in fertility and an increase in the mortality of EPNs [215]. Acetamiprid and thiamethoxam show weaker synergism with EPNs [216]. Chlorpyrifos is an organophosphate insecticide that is more toxic than acetamiprid toward *S. feltiae* [217]. It was also demonstrated that this EPN species was more sensitive to insecticides than were other species, e.g., *H. bacteriophora*, *S. arenarium*, and *S. kraussei* [205,217]. An EPN species that was not adversely affected by chlorpyrifos and the pyrethroid insecticide cypermethrin was *S. carpocapsae* [209]. Another group of insecticides are the carbamates, among which are carbosulfan and carbofuran. These agents did not affect the pathogenicity of the nematodes studied, but carbofuran killed more nematodes than did carbosulfan [218]. Either of the above-mentioned carbamates and pirimicarb can be used in IPM together with EPNs [210,218]. Macrocyclic lactones include abamectin. Like lufenuron, a benzoylurea insecticide also used to control fleas, fungi, and nematodes in mammals, abamectin adversely affects *H. bacteriophora* [210]. Another agent from the macrocyclic lactone insecticides is emamectin benzoate, which is effective against the spotted wing fly (*D. suzukii*) in berry crops. This insecticide has also exhibited adverse effects on the infectivity of *S. carpocapsae* [209].

### 5.4. Fertilizers

Fertilizer usage is also one of the agrochemical treatments which may impact EPNs. Excessive use of inorganic fertilizers contributes to increased soil salinity and accumulation of heavy metals and nitrates [219,220]. In saline soil, EPN movement is hindered and their ability to find and identify hosts is limited [221].

Long-term exposure to high concentrations of inorganic fertilizers inhibited the infectivity and reproduction of nematodes, whereas 1-day exposure of EPNs to the fertilizer increased infectivity. Interspecific differences in EPNs’ sensitivity to fertilizers were also observed. *H. bacteriophora* was more sensitive to negative impacts of fertilizers than were *S. feltiae* and *S. arenarium*. In field trials, an organic fertilizer increased *S. feltiae* density, whereas NPK fertilizer decreased nematode density regardless of manure applications [222].

Analysis of the effect of various fertilizers on EPNs showed that all the fertilizers tested led to the death of the studied nematodes. On the other hand, the highest toxicity to *H. bacteriophora* and *S. feltiae* was achieved by nitrogen–phosphorus and diammonium phosphate fertilizers [223]. In other research, it was proven that the infectivity of EPNs to insects was lower in soil treated with phosphorus fertilizer than in soil treated with potassium, NPK, or nitrogen fertilizers [224]. The combined application of inorganic fertilizers with NPK and Mikrovit (MgO, 2.33%; Cu, 0.25%; Mn, 0.17%; Zn, 0.18%; B, 0.015%; Fe, 0.23%; Mo, 0.01%), against a background of liming was conducive to greater activity of nematodes in infesting insects, while it adversely affected the reproduction of EPNs [225]. The reproduction of nematodes was also depressed by NPK fertilizer in a time-dependent manner. It was shown that, after 20 and 40 days of exposure to NPK fertilizer, there was a strong decrease in the intensity of infestation. The use of higher doses of mineral fertilizers resulted in a decrease in the reproduction of nematodes developing within the host, which led to a decrease in the number of nematodes in the soil. The exceptions were potassium nitrate and manure, which stimulated the reproduction of the nematodes [129].

### 5.5. Essential Oils

Essential oils, due to their broad spectrum biological of activities (bactericidal, virucidal, fungicidal, antiparasitic, insecticidal, therapeutic, and cosmetic), are currently used in the pharmaceutical, sanitary, cosmetic, food, and agricultural industries [226,227,228,229,230]. In plant protection, they are increasingly used as natural pesticides and as a rational element of IPM [231].

In research on the toxicity toward EPNs of essential oils, thyme, cinnamon, clove, and garlic oils have been shown to exert the most negative effects, causing 100% mortality of *S. feltiae* and *H. bacteriophora* at a 1% concentration [231]. Pine oil led to mortality of 50% of *H. bacteriophora*, while lemongrass oil killed more *S. feltiae* individuals than *H. bacteriophora* juveniles. Spearmint, cedarwood, eucalyptus, and rosemary oils had no significant effect on nematode mortality [231]. The most promising oil seems to be eucalyptus oil. It is also an effective pesticide against plant pests [232]. These properties indicate that this oil can be used in IPM in combination with EPN-based preparations [231].

In order to reduce EPN stress caused by the drying out or UV irradiation of surfactants applied to the above-ground parts of plants, the possibility of using coconut oil and olive oil as adjuvants in EPN preparations has been investigated [233].

## 6. Interspecies Relationships

For EPNs, hosts are essential biotic factors. The morphological, anatomical, and physiological properties of the insect enable nematodes to find, infest, and develop in its body cavity. For example, an increasing concentration of host excretions, which is a signal of the nearby presence of the host, causes an increase in the activity of infective juveniles [234].

EPNs have to share soil with a huge diversity of living organisms and microorganisms in addition to the pests against which they are targeted. Numerous positive and negative interactions between EPNs and soil organisms other than their hosts have been documented [235,236].

### 6.1. Hosts

Natural invasions of EPNs in the environment in the form of nematode-infested insects in ecosystems are rarely observed. This is due to the rapid development of the nematode population in the host and the rapid destruction of its body. Of the dozens of beetle species in which the larvae are sensitive to the nematode *S. carpocapsae*, only a few have been successfully studied with respect to natural invasion, namely, *Zabrus tenebrioides* Goeze (Coleoptera: Carabidae) (the wheat ground beetle) [237], *Agriotes lineatus* Linnaeus (Coleoptera: Elateridae) (the lined click beetle) [238], *Melolontha melolontha* Linnaeus (Coleoptera: Scarabaeidae) (the cockchafer), *Drosophila suzukii* Matsumura (Diptera: Drosophilidae), and *Melolontha hippocastani* Fabricius (Coleoptera: Scarabaeidae) (the forest cockchafer) [10,239]. It has also been possible to isolate *S. feltiae* from flies [240] and *Heterorhabditis heliothidis* Khan, Brooks and Hirschmann (Rhabditida: Heterorhabditidae) from *Helicoverpa zea* Boddie (Lepidoptera: Noctuoidea) (the corn earworm) [241]. *H. bacteriophora* has also been found in *Helicoverpa punctigera* Wallengren (Lepidoptera: Noctuoidea) (the native budworm) [237] and *Ceratitis capitata* Wiedemann (Diptera: Tephritidae). It has also been shown that *S. kraussei* infested *Cephalcia abietis* Linnaeus (Hymenoptera: Pamphiliidae) (the false spruce webworm) under natural conditions [242,243].

The wide host range of EPNs allows for the biological control of many phytophagous species. To date, EPNs have been used in the control of ground flies (Diptera: Sciaridae) in greenhouse crops [244] and mushroom crops [245]; weevils (Coleoptera: Curculionidae) in greenhouses and nurseries of ornamental and forest trees [246,247,248]; beetles (Coleoptera: Scarabaeidae) on urban lawns, golf courses, and forest nurseries [249]; and geometer moths (Lepidoptera: Geometridae), *Leptinotarsa decemlineata* Say (Coleoptera: Chrysomelidae) (the Colorado potato beetle), and sawflies (Hymenoptera: Symphyta), as well as in the control of a number of tree pests that pupate in the soil [250,251]. However, certain EPNs are specialists that usually target one type of insect [108]. For example, *Steinernema diaprepesi* Nguyen and Duncan (Rhabditida: Steinernematidae) targets the larval stages of the *Diaprepes abbreviatus* Linnaeus (Coleoptera: Curculionidae) (the root weevil) [252,253] and *Steinernema neocurtillae* Nguyen and Smart (Rhabditida: Steinernematidae) targets only *Neocurtilla hexadactyla* Perty (Ortoptera: Gryllotalpidae) (mole crickets) [70,83].

Despite the association of EPN hosts with particular insect orders, as shown above, different sensitivity to EPNs is commonly shown. The larvae of moths or butterflies (Lepidoptera) are most often infected. Beetles (Coleoptera) (especially adults) and flies (Diptera: Muscidae) are more resistant to EPNs [254]. The larvae of ladybirds *Hippodamia convergens* Crotch (Coleoptera: Coccinellidae) (the convergent ladybird) and golden-eyed beetles *Hemerobius* spp. (Neuroptera: Hemerobiidae) and *Chrysopa* spp. (Neuroptera: Chrysopidae) were shown to be resistant to EPN infestation [237]. The development of nematodes in a host’s body is also influenced by the developmental stage of the insect. Nematodes thrive best in the larval stages, less so in pupal and mature imago forms [255,256]. In addition to the developmental stage of the insect, the development of EPNs is also influenced by the level of nutrition of the host. It has been shown that in starved host insects, the development of nematodes slows down [257]. It has also been found that as the density of nematodes in the host increases, the number of generations decreases [258].

Among the morphological features of insects that facilitate the invasion of nematodes are the size of the natural openings (the mouth, anus, and spiracles) and the structure of the outer sheath [259,260]. The ability of infective juveniles to penetrate an insect also depends on the period of contact of the nematodes with the host or its feces. The longer the duration of contact, the lower the chance of penetrating the insect [261,262]. The development of nematodes and their density in the environment depend, among other things, on the number of potential insect hosts in the environment: the more insects available, the better the chances of survival of EPN populations [115]. The reproduction rate of EPNs also depends on the host’s body weight. Nematode reproduction is much greater in a host with a higher body weight [263]. The reproductive capacity of nematodes is also affected by the origin of the infective form. It was found that the reproductive rate was higher after several passages trough the same host species than with the use of juveniles where the previous generations had undergone development in another host species [256,264].

### 6.2. Mites

It has been observed that a large number of nematophagous arthropods attack and eat infective juveniles, whereas smaller arthropod larvae only damage them. The introduction of EPNs into the soil stimulates the development of populations of nematophagous arthropods, which, in turn, effectively reduce the number of nematodes. It was shown that certain species of mites (*Gamasellodes vermivorax* Walter (Mesostigmata: Ascidae) and *Alycus roseus* Koch (Trombidiformes: Alycidae)) feed on IJ EPNs [265]. Mites that do not eat EPNs have been observed too. Studies have shown that *Sancassania polyphyllae* Zachvatkin (Acari: Acaridae) do not consume infective juveniles in the soil [266].

There are also mites in the soil which are killed by the symbiotic EPN bacterium *Photorhabdus luminescens* (Thomas and Poinar) Boemare, Akhurst and Mourant (Enterobacterales: Morganellaceae). *P. luminescens* resulted in a significantly higher mortality rate of adult *Tetranychus truncatus* Ehara (Acari: Tetranychidae) than *Pseudomonas aeruginosa* (Schroeter) Migula (Pseudomonadales: Pseudomonadaceae) [267].

### 6.3. Springtails

Springtails (Collembola) are organisms that mainly inhabit the soil and litter layers, which are also the environment of IJs of EPNs. These arthropods pose a major threat to them. It was shown that springtail species—*Sinella curviseta* Brook (Entomobryomorpha: Entomobryidae), *Folsomia candida* Willem (Entomobrymorpha: Isotomidae), and *Hypogastrura scotti* Yosii (Poduromorpha: Hypogastruridae)—consumed significant numbers of IJs [265,266]. Moreover, the presence of EPNs in the soil increases the abundance of springtails [268]. The ability of *F. candida* and *Sinella caeca* Zhao (Entomobryomorpha: Entomobryidae) to eat three species of entomopathogenic nematodes (*S. carpocapsae*, *S. feltiae*, and *S. glaseri*) and to reduce the infectiveness of nematode applications against insects was investigated. It was shown that as few as five springtails are enough to reduce the invasiveness of EPNs against the insect [269].

### 6.4. Nematodes

Competition is a natural factor in the struggle for existence in the soil. Organisms that inhabit an ecosystem often have to compete in many ways for food, territory, or survival. The biological activity of nematodes is also influenced by both interspecific competition among nematodes and the attractiveness of the host. Scavenger free-living nematodes such as *Oscheius* spp. (Rhabditida: Rhabditidae) are able to displace EPNs from their place of occurrence [270]. By studying the simultaneous introduction of infective juveniles of both *S. feltiae* and *H. bacteriophora* into the same environment, it was shown that, in the absence of insect hosts in the soil, dispersal is characterized very largely by randomness. On the other hand, after the introduction of potential hosts (*G. mellonella*, *Tenebrio molitor* Linnaeus (Coleoptera: Tenebrionidae), and *Tribolium confusum* Jacquelin du Val (Coleoptera: Tenebrionidae)) into the environment, there was a change in the speed and direction of movement of the nematode juveniles. In response to the introduction of one host insect species into the soil environment, an increase in the activity of both *S. feltiae* and *H. bacteriophora* was demonstrated. Both nematode species showed the ability to recognize and select the more attractive host. *S. feltiae* and *H. bacteriophora* accumulated considerably in the vicinity of *G. mellonella*, less so in the vicinity of *T. molitor*, and least near *T. confusum*. Simultaneous introduction of the two nematode species into the soil resulted in the faster movement of *S. feltiae* than *H. bacteriophora* toward the hosts. As the contact time increased, the number of infective juveniles near the insect increased. In cross-inoculation, *S. feltiae* showed the same food preference as in single-species inoculation. In cross-inoculation, *H. bacteriophora* was directed to the regions of the host that were less attractive to and therefore less affected by the competitor species (*S. feltiae*) [271].

The density of nematodes in the soil can be affected by the presence of other soil fauna. Among the nematodes found in the soil environment, there are nematophagous nematodes, which reduce the number of EPNs. It was found that, during the 8-week period after the introduction of infective *S. carpocapsae* juveniles into the soil, their number systematically decreased, while the number of predatory nematodes (Mononchida) and saprophagous nematodes (Rhabditida) definitely increased. This showed that EPNs provide a food base for other trophic groups of nematodes [144]. Other research showed that *Mononchoides longicaudatus* Khera (Rhabditida: Diplogastridae) feed on the infective juveniles of *S. carpocapsae* and *H. bacteriophora* [272].

There are also nematodes in the soil environment that are pests of plants, for instance, *Meloidogyne* spp. (Tylenchida: Heteroderidae). Plant-pathogenic nematodes suppress entomopathogenic nematodes and factors from EPNs can repel plant-pathogenic nematodes [273,274].

### 6.5. Fungi

Another group of organisms that reduce EPN populations are fungi. More than 150 species of predatory and endoparasitic fungi have been shown to reduce the population of EPNs in the natural soil environment [275]. Predatory fungi form two types of traps (sticky and ring traps). Sticky traps of *Arthrobotrys oligospora* have hyphae which form a network of sticky meshes. EPN infective juveniles adhere to the mesh of the web and form a food base for the fungus. The ring trap of *Dactylaria candida* is made up of several mycelial cells. The ring is sensitive to touch and movement and can tighten when a nematode is inside. Traps of this type can also be adapted to the diameters of nematodes; as a nematode passes through the ring, it constricts when the nematode touches the inner walls of the ring [124]. The study showed high infectivity of *Arthrobotrys musiformis* and *A. oliogospora* against EPNs. *A. musiformis* is more dangerous because it killed more nematode species used in the experiment [276].

Research on predatory fungi (*Monacrosporium ellipsosporum* and *A. oligospora*) showed that, within 72 h, these fungi attacked and destroyed nematodes in vitro. In the case of the endoparasitic fungus *Drechmeria coniospora*, it was observed that the spores of the fungus infected EPN juveniles in the soil, colonized them, but did not infect individuals of the first generation of nematodes developing within the host [277].

Among fungi, there are also species that can act similarly to EPNs in their activity toward pests. Synergism was shown to exist between these two groups [142]. Research used entomopathogenic fungi (*Beauveria bassiana* and *Paecilomyces farinosus*) and EPNs (*S. carpocapsae* and *H. bacteriophora*) to protect peas from the pea leaf weevil, *Sitona lineatus* Linnaeus (Coleoptera: Curculionidae). It was shown that there were significantly fewer live larvae of the pest on plots with both fungi and nematodes present [278].

### 6.6. Bacteria

The life processes of EPNs are also affected by interactions with soil bacteria. Studies have shown that *Serratia marcescens* Bizio (Enterobacterales: Yersiniaceae) adversely affects nematodes’ penetration into the host’s body, limits their growth, and inhibits reproduction [279]. After entering the host, the nematodes are exposed to its intestinal microflora, which is unfavorable to symbiotic bacteria EPNs, reducing the infectivity of the parasite [280]. Other bacteria associated with nematodes are *Paenibacillus* sp. (Bacillales: Paenibacillaceae). These bacteria adhere to the nematode’s cuticle and reproduce in the body of the insect infected with the nematode. A phoretic relationship between *Paenibacillus nematophilus* and EPNs (*Heterorhabditis* spp.) has been documented. It depends on the fact that the bacteria use nematodes as a means of transport into the insect’s body without doing them any harm [235,281,282].

## 7. Summary

Entomopathogenic nematodes are naturally occurring organisms in the environment. A wide host circle and adaptability to a diverse range of soil environments make EPNs excellent agents for biological control of insect pests. Like other organisms, EPNs are affected in various ways by environmental soil pollutants in the form of heavy metals, petroleum substances, pesticides, fertilizers, and even essential oils, in response to which these organisms will struggle. Continuous development of plant protection methods allows for even more effective use of the insecticidal and adaptive abilities of EPNs (Table 1).

## Figures and Tables

**Table 1:** Environmental factors influencing entomopathogenic nematodes.

Environmental Factor	Value	Significance for EPNs
Abiotic Factors		
Soil texture	Textural classes (e.g., sandy, loam, and sandy loam)	Movement, infectivity, and survivability [48,99,118,119,120,121,122,123,124,125,126,127,129,130,131,132]
Moisture	25–40%	Under optimal conditions, nematodes move actively in the soil [48]
	>50%	Significant decreases in nematode motility and survival occur [48]
Temperature	13–25 °C	Optimal temperature range for development and biological activity [149]
	<−6 °C (nonencapsulated nematodes)	Below these values, death of the organisms occurs [154]
	<−32 °C (capsulated nematodes)	
	>32 °C	Above this value, biological activity and survival decrease significantly [146,156]
	>37 °C	Above this value, there is death of the organisms [157,158]
Soil pH	5.5–10	Optimal value range for survival and infectivity [163,164]
	<4	Significant deterioration in survival and infectivity [164]
Radiation	Wavelength of 366 nm	No noticeable changes [166,167]
	Wavelength of 254 nm, with an exposure time of >1.75 min	Reduced pathogenicity and reproduction and increased mortality [166,167]
	Gamma radiation	Improved biological effectiveness against plant pests [169,170]
Soil aeration	1–20%	High survivability and density [164,173]
	Low oxygen content	Significant increase in mortality [171,172]
Metal ions	Cd(II) and Pb(II)	Low concentrations of these metals result in an increase in infectivity, whereas higher concentrations result in a decrease in infectivity and speed of movement and an increase in mortality [187,191]
	Co(II), Cu(II), Mg(II), and Zn(II)	Toxic effects in combination with Cd(II) and Pb(II) [180]
	Mg(II)	Reduction in metal triad toxicity from Cd(II) or high nematode mortality [180]
	Mn(II)	Exhibits the lowest toxicity toward EPNs of all the metals tested [180]
Petroleum	Crude oil, diesel, engine oil, and gasoline	Reduction in pathogenicity and reproduction of EPNs [200,202,203,204]
Pesticides	E.g., thiamethoxam, chlorpyrifos, and carbosulfan	Reduced pathogenicity, reproduction, and invasiveness [205,207,208]
Fertilizers	Nitrates and phosphates	Cause an increase in mortality and reduce reproduction, infectivity, and speed of movement in soil [129,221,224,225]
Essential oils	Thyme oil, cinnamon oil, clove oil, and garlic oil	Significant increase in mortality [231]
	Spearmint oil, cedarwood oil, eucalyptus oil, and rosemary oil	No significant effect on nematode mortality [231,232]
Biotic Factors		
Hosts	Increased concentration of insect excreta in the environment	Improved host localization [115,255,256]
	Host larval stage	Greatest infectivity [255,256]
	Pupal or imago stage	Significantly reduced infectivity [255,256]
	Resistance to EPNs	The more resistant the insect host species, the lower the chance of the EPN population expanding [237,254]
Mites	*Gamasellodes vermivorax* and *Alycus roseus*	Decreased numbers of EPNs in the soil [265,266]
Springtails	*Sinella curviseta*, *Folsomia candida*, and *Hypogastrura scotti*	Decreased numbers of EPNs in the soil and reduction in infectivity [265,266,269]
Nematodes	*Oscheius* spp.	Displace EPNs form the place of occurrence [270]
	Other species of EPNs	Increased biological activity [271]
	Nematophagous nematodes	Reduced the number of EPNs [144,272]
	Plant-pathogenic nematodes	Suppressed EPNs and antagonistic effect [273,274]
Fungi	Predatory fungi	Reduced populations of nematodes in the soil [124,275,276,277]
	Entomopathogenic fungi	Synergistic effect [142,278]
Bacteria	*Serratia marcescens*	Limits growth and reproduction [279]
	Host microbiome	Reduced infectivity [280]
	*Paenibacillus nematophilus*	Phoretic relationship [235,281,282]

## Data Availability

Dataset is available on reasonable request from the corresponding author.
